# *rac-*Methyl 3-(2-meth­oxy­phen­yl)-1-phenyl-3,3a,4,9b-tetra­hydro-1*H*-chromeno[4,3-*c*]isoxazole-3a-carboxyl­ate

**DOI:** 10.1107/S1600536812021356

**Published:** 2012-06-02

**Authors:** S. Paramasivam, J. Srinivasan, P. R. Seshadri, M. Bakthadoss

**Affiliations:** a Post Graduate and Research Department of Physics, Agurchand Manmull Jain College, Chennai 600 114, India; b Department of Organic Chemistry, University of, Madras, Guindy Campus, Chennai 600 025, India.

## Abstract

The title compound, C_25_H_23_NO_5_, comprising two stereogenic carbon atoms of the same configuration, crystallizes in a centrosymmetric space group as a racemate. The six-membered pyran ring and the five-membered isoxazole ring adopt sofa and twisted conformations, respectively. The dihedral angle between the benzene ring and the mean plane through the near coplanar atoms of the pyran ring is 10.73 (7)°. The crystal structure features C—H⋯O hydrogen bonds.

## Related literature

For the biological activity of the title compound, see: Eddington *et al.* (2002 ▶); Mullen *et al.* (1988 ▶); Kashiwada *et al.* (2001 ▶); Caine (1993 ▶). For N-atom hybridization, see: Beddoes *et al.* (1986 ▶). For related structures, see: Kanchanadevi *et al.* (2011 ▶); Swaminathan *et al.* (2012 ▶). For conformational analysis and puckering parameters, see: Cremer & Pople (1975 ▶).
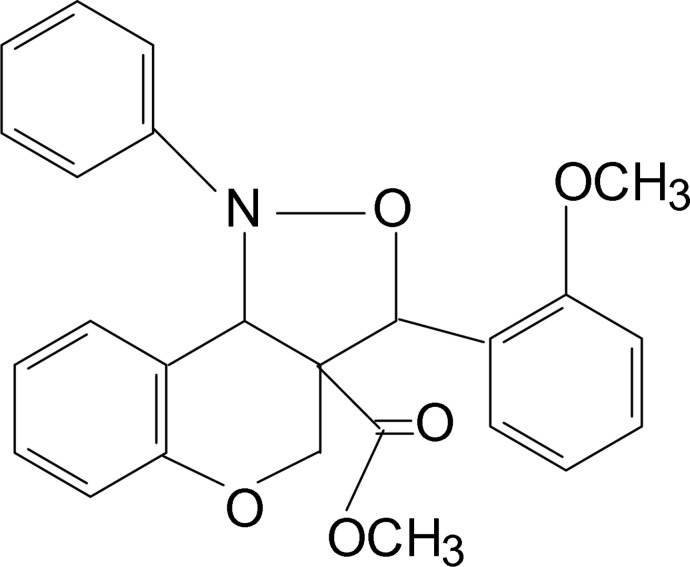


## Experimental

### 

#### Crystal data

C_25_H_23_NO_5_
*M* *_r_* = 417.44
Monoclinic, 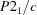

*a* = 18.3791 (7) Å
*b* = 15.2466 (6) Å
*c* = 7.7235 (3) Å
β = 90.514 (2)°
*V* = 2164.18 (15) Å^3^
*Z* = 4
Mo *K*α radiation
μ = 0.09 mm^−1^
*T* = 298 K
0.20 × 0.15 × 0.10 mm

#### Data collection

Bruker SMART APEXII area-detector diffractometer
20989 measured reflections
5478 independent reflections
3614 reflections with *I* > 2σ(*I*)
*R*_int_ = 0.036

#### Refinement

*R*[*F* ^2^ > 2σ(*F* ^2^)] = 0.050
*wR*(*F* ^2^) = 0.159
*S* = 0.96
5478 reflections
280 parameters
H-atom parameters constrained
Δρ_max_ = 0.24 e Å^−3^
Δρ_min_ = −0.16 e Å^−3^

### 

Data collection: *APEX2* (Bruker, 2008 ▶); cell refinement: *SAINT* (Bruker, 2008 ▶); data reduction: *SAINT*; program(s) used to solve structure: *SHELXS97* (Sheldrick, 2008 ▶); program(s) used to refine structure: *SHELXL97* (Sheldrick, 2008 ▶); molecular graphics: *ORTEP-3* (Farrugia, 1997 ▶) and *PLATON* (Spek, 2009 ▶); software used to prepare material for publication: *SHELXL97* (Sheldrick, 2008 ▶), *PLATON* and *publCIF* (Westrip, 2010 ▶).

## Supplementary Material

Crystal structure: contains datablock(s) I, global. DOI: 10.1107/S1600536812021356/kp2411sup1.cif

Structure factors: contains datablock(s) guru2. DOI: 10.1107/S1600536812021356/kp2411Isup2.hkl

Supplementary material file. DOI: 10.1107/S1600536812021356/kp2411Isup3.cml

Additional supplementary materials:  crystallographic information; 3D view; checkCIF report

## Figures and Tables

**Table 1:** Hydrogen-bond geometry (Å, °)

*D*—H⋯*A*	*D*—H	H⋯*A*	*D*⋯*A*	*D*—H⋯*A*
C7—H7⋯O4	0.98	2.33	2.803 (2)	109
C15—H15⋯O3^i^	0.93	2.42	3.285 (3)	155

Symmetry code: (i) 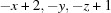.

**Fig. 1.**
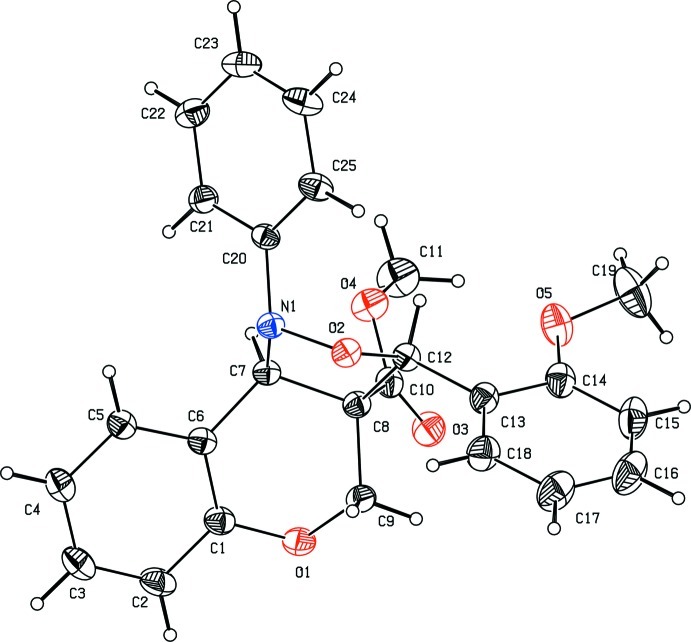
The molecular structure of the title compound showing the atom-numbering scheme and displacement ellipsoids drawn at the 20% probability level.

**Fig. 2.**
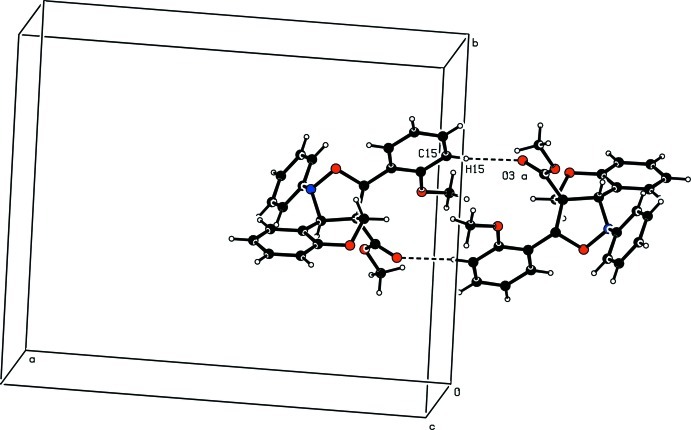
The crystal packing of the title compound. Hydrogen bonds are shown by dashed lines.

## supplementary crystallographic information

### Comment

Isoxazole derivative exhibit anti-consulvant (Eddington *et al.*, 2002) and anti-fungal (Mullen *et al.*, 1988) activities, whereas benzopyran and chromenopyrrole derivatives are used in the treatment of impulsive-disorder disease (Caine, 1993) and exhibit anti-HIV activities (Kashiwada *et al.*, 2001). On this grounds, the title compound was chosen for X-ray structure analysis (Fig.1).

The pyran ring (O1/C1/C6—C9) adopts a sofa conformation with the puckering parameters (Cremer & Pople, 1975) being q_2_=0.478 (2) Å, q_3_=0.230 (2) Å, Q_T_=0.531 (2) Å and the five membered ring isoxazole (O2/N1/C7/C8/C12) adopts a twisted conformation with puckering parameters (Cremer & Pople, 1975) being q_2_=0.429 (1) Å and Φ_2_=165.9 (2)°. The dihedral angle between the pyran and the benzene ring (C1—C6) is 10.73 (7)°. The dihedral angle between the chromeno ring (fusion of benzene and pyran rings) and isoxazole ring is 57.52 (5)°.

In the chromenoisoxazole moiety, the dihedral angle between the benzene and isoxazole ring is 56.88 (6)°. The dihedral angle between the pyran and isoxazole ring is 56.27 (6)°. The sum of the bond angles around N1 [334.55 (39)°] indicates sp3 hybridization (Beddoes *et al.*,1986). The unit cell contains no residual solvent accessible voids, if the voids in the dry crystals ever contained solvent, though generally solvent loss from organic crystals is associated with either a total loss of crystallinity or at least a degradation of the crystal quality. In this case the crystals remained glass-clear.

The geometric parameters of the title compound (Fig. 1) agree well with the reported similar structures (Kanchanadevi *et al.*, 2011; Swaminathan *et al.*, 2012).

The molecular structure is stabilized by C— H··· O intramolecular interactions and the crystal packing is via C— H··· O hydrogen bonds(Table 1, Fig. 2).

### Experimental

A mixture of (*E*)-methyl 2-((2-formylphenoxy)methyl)-3-(2-methoxyphenyl)acrylate (2 mmol, 0.65 g) and *N*-phenylhydroxylamine (3 mmol, 0.33 g) in ethanol (10 mL) was refluxed for 6 h. After the completion of the reaction as indicated by TLC, the reaction mixture was concentrated and the resulting crude mass was diluted with water (15 mL) and extracted with ethyl acetate (3 × 15 mL). The combined organic layer was washed with brine (3 × 15 mL) and dried over anhydrous Na_2_SO_4_, solvent was removed under reduced pressure. The crude mass was purified by column chromatography on silica gel (Acme 100–200 mesh), using ethyl acetate-hexane (0.5: 9.5) to afford the pure compound as a colourless solid in 91% yield.

### Refinement

Hydrogen atoms were positioned geometrically and allowed to ride on their parent atoms, with C—H = 0.93 - 0.97 Å and *U*_iso_(H) = 1.5*U*_eq_(C) for methyl H atoms and 1.2 *U*_eq_(C) for other H atoms.

### Crystal data

**Table:**

C_25_H_23_NO_5_	*F*(000) = 880
*M**_r_* = 417.44	*D*_x_ = 1.281 Mg m^−^^3^
Monoclinic, *P*2_1_/*c*	Mo *K*α radiation, λ = 0.71073 Å
Hall symbol: -P 2ybc	Cell parameters from 5478 reflections
*a* = 18.3791 (7) Å	θ = 1.7–28.6°
*b* = 15.2466 (6) Å	µ = 0.09 mm^−^^1^
*c* = 7.7235 (3) Å	*T* = 298 K
β = 90.514 (2)°	Block, colourless
*V* = 2164.18 (15) Å^3^	0.20 × 0.15 × 0.10 mm
*Z* = 4	

### Data collection

**Table:**

Bruker SMART APEXII area-detector diffractometer	3614 reflections with *I* > 2σ(*I*)
Radiation source: fine-focus sealed tube	*R*_int_ = 0.036
Graphite monochromator	θ_max_ = 28.6°, θ_min_ = 1.7°
ω and φ scans	*h* = −23→24
20989 measured reflections	*k* = −20→17
5478 independent reflections	*l* = −10→10

### Refinement

**Table:**

Refinement on *F*^2^	Primary atom site location: structure-invariant direct methods
Least-squares matrix: full	Secondary atom site location: difference Fourier map
*R*[*F*^2^ > 2σ(*F*^2^)] = 0.050	Hydrogen site location: inferred from neighbouring sites
*wR*(*F*^2^) = 0.159	H-atom parameters constrained
*S* = 0.96	*w* = 1/[σ^2^(*F*_o_^2^) + (0.0739*P*)^2^ + 0.7112*P*] where *P* = (*F*_o_^2^ + 2*F*_c_^2^)/3
5478 reflections	(Δ/σ)_max_ = 0.031
280 parameters	Δρ_max_ = 0.24 e Å^−^^3^
0 restraints	Δρ_min_ = −0.16 e Å^−^^3^

### Special details

**Table:**

Geometry. All esds (except the esd in the dihedral angle between two l.s. planes) are estimated using the full covariance matrix. The cell esds are taken into account individually in the estimation of esds in distances, angles and torsion angles; correlations between esds in cell parameters are only used when they are defined by crystal symmetry. An approximate (isotropic) treatment of cell esds is used for estimating esds involving l.s. planes.
Refinement. Refinement of F^2^ against ALL reflections. The weighted R-factor wR and goodness of fit S are based on F^2^, conventional R-factors R are based on F, with F set to zero for negative F^2^. The threshold expression of F^2^ > 2sigma(F^2^) is used only for calculating R-factors(gt) etc. and is not relevant to the choice of reflections for refinement. R-factors based on F^2^ are statistically about twice as large as those based on F, and R- factors based on ALL data will be even larger.

### Fractional atomic coordinates and isotropic or equivalent isotropic displacement parameters (Å^2^)

**Table:**

	*x*	*y*	*z*	*U*_iso_*/*U*_eq_	
O2	0.70764 (6)	0.09270 (7)	0.44631 (14)	0.0483 (3)	
O3	0.86302 (8)	−0.14409 (10)	0.4169 (2)	0.0700 (4)	
O1	0.73699 (8)	−0.14734 (9)	0.68811 (17)	0.0632 (4)	
O4	0.79918 (8)	−0.10360 (10)	0.18406 (17)	0.0667 (4)	
N1	0.65661 (8)	0.03750 (9)	0.35751 (17)	0.0434 (3)	
O5	0.91863 (8)	0.05907 (11)	0.3191 (2)	0.0835 (5)	
C8	0.76235 (9)	−0.04489 (11)	0.4528 (2)	0.0456 (4)	
C20	0.64690 (9)	0.06345 (11)	0.1815 (2)	0.0439 (4)	
C7	0.68195 (9)	−0.05364 (10)	0.3918 (2)	0.0426 (4)	
H7	0.6806	−0.0866	0.2829	0.051*	
C25	0.66789 (12)	0.14637 (12)	0.1260 (2)	0.0574 (5)	
H25	0.6940	0.1831	0.1998	0.069*	
C6	0.63369 (10)	−0.09929 (10)	0.5208 (2)	0.0456 (4)	
C12	0.77738 (9)	0.05377 (11)	0.4147 (2)	0.0463 (4)	
H12	0.7899	0.0610	0.2924	0.056*	
C5	0.55870 (10)	−0.09931 (11)	0.4991 (2)	0.0512 (4)	
H5	0.5380	−0.0670	0.4092	0.061*	
C1	0.66301 (11)	−0.14728 (11)	0.6568 (2)	0.0532 (4)	
C13	0.83338 (11)	0.09872 (11)	0.5257 (2)	0.0549 (4)	
C10	0.81416 (10)	−0.10354 (11)	0.3522 (2)	0.0503 (4)	
C9	0.76871 (11)	−0.06416 (13)	0.6460 (2)	0.0565 (5)	
H9A	0.8196	−0.0642	0.6801	0.068*	
H9B	0.7444	−0.0183	0.7104	0.068*	
C14	0.90540 (11)	0.10027 (12)	0.4729 (3)	0.0640 (5)	
C21	0.60827 (10)	0.00969 (12)	0.0687 (2)	0.0529 (4)	
H21	0.5934	−0.0457	0.1044	0.064*	
C4	0.51420 (12)	−0.14634 (13)	0.6085 (3)	0.0635 (5)	
H4	0.4640	−0.1452	0.5929	0.076*	
C24	0.64973 (14)	0.17402 (15)	−0.0393 (3)	0.0731 (6)	
H24	0.6633	0.2299	−0.0749	0.088*	
C22	0.59180 (12)	0.03867 (15)	−0.0977 (2)	0.0650 (5)	
H22	0.5666	0.0020	−0.1735	0.078*	
C18	0.81593 (14)	0.13951 (14)	0.6799 (3)	0.0714 (6)	
H18	0.7678	0.1398	0.7162	0.086*	
C23	0.61227 (14)	0.12089 (16)	−0.1515 (3)	0.0734 (6)	
H23	0.6008	0.1401	−0.2627	0.088*	
C2	0.61876 (13)	−0.19588 (13)	0.7667 (3)	0.0679 (6)	
H2	0.6391	−0.2285	0.8565	0.082*	
C3	0.54475 (14)	−0.19499 (15)	0.7408 (3)	0.0732 (6)	
H3	0.5149	−0.2275	0.8133	0.088*	
C15	0.95828 (13)	0.14082 (15)	0.5751 (4)	0.0845 (8)	
H15	1.0066	0.1413	0.5400	0.101*	
C17	0.86869 (17)	0.17994 (18)	0.7813 (4)	0.0917 (8)	
H17	0.8562	0.2067	0.8851	0.110*	
C11	0.84555 (15)	−0.15710 (19)	0.0768 (3)	0.0894 (8)	
H11A	0.8301	−0.1523	−0.0419	0.134*	
H11B	0.8423	−0.2172	0.1131	0.134*	
H11C	0.8950	−0.1374	0.0881	0.134*	
C16	0.93901 (18)	0.18001 (17)	0.7273 (4)	0.0971 (9)	
H16	0.9745	0.2071	0.7951	0.116*	
C19	0.98762 (16)	0.0700 (2)	0.2409 (5)	0.1113 (11)	
H19A	0.9888	0.0379	0.1340	0.167*	
H19B	1.0249	0.0483	0.3175	0.167*	
H19C	0.9958	0.1311	0.2183	0.167*	

### Atomic displacement parameters (Å^2^)

**Table:**

	*U*^11^	*U*^22^	*U*^33^	*U*^12^	*U*^13^	*U*^23^
O2	0.0549 (7)	0.0414 (6)	0.0488 (6)	0.0020 (5)	−0.0010 (5)	−0.0077 (5)
O3	0.0567 (8)	0.0680 (9)	0.0852 (10)	0.0145 (7)	−0.0006 (7)	0.0109 (7)
O1	0.0704 (9)	0.0569 (8)	0.0621 (8)	0.0013 (6)	−0.0071 (6)	0.0185 (6)
O4	0.0702 (9)	0.0720 (9)	0.0579 (8)	0.0221 (7)	0.0024 (6)	−0.0124 (6)
N1	0.0489 (8)	0.0390 (7)	0.0423 (7)	0.0010 (6)	−0.0015 (6)	0.0005 (5)
O5	0.0618 (10)	0.0805 (11)	0.1085 (13)	−0.0042 (8)	0.0218 (9)	−0.0086 (9)
C8	0.0499 (9)	0.0412 (8)	0.0457 (8)	0.0014 (7)	−0.0021 (7)	0.0010 (7)
C20	0.0470 (9)	0.0433 (8)	0.0416 (8)	0.0052 (7)	0.0068 (7)	0.0026 (6)
C7	0.0498 (9)	0.0373 (8)	0.0406 (7)	0.0020 (6)	0.0002 (6)	−0.0001 (6)
C25	0.0795 (13)	0.0439 (9)	0.0490 (9)	0.0004 (9)	0.0094 (9)	0.0005 (7)
C6	0.0564 (10)	0.0357 (8)	0.0448 (8)	−0.0012 (7)	0.0018 (7)	−0.0013 (6)
C12	0.0505 (10)	0.0412 (8)	0.0472 (8)	−0.0001 (7)	0.0017 (7)	−0.0032 (7)
C5	0.0580 (11)	0.0434 (9)	0.0522 (9)	−0.0029 (8)	0.0040 (8)	−0.0024 (7)
C1	0.0644 (12)	0.0420 (9)	0.0532 (9)	−0.0008 (8)	0.0001 (8)	0.0041 (7)
C13	0.0583 (11)	0.0423 (9)	0.0641 (11)	−0.0051 (8)	−0.0055 (9)	−0.0011 (8)
C10	0.0480 (10)	0.0397 (8)	0.0634 (10)	0.0003 (7)	0.0004 (8)	0.0033 (7)
C9	0.0599 (11)	0.0585 (11)	0.0510 (9)	−0.0046 (9)	−0.0081 (8)	0.0068 (8)
C14	0.0602 (12)	0.0460 (10)	0.0859 (14)	−0.0023 (9)	−0.0037 (10)	0.0049 (10)
C21	0.0557 (11)	0.0529 (10)	0.0502 (9)	−0.0014 (8)	−0.0035 (8)	0.0028 (8)
C4	0.0636 (12)	0.0572 (11)	0.0698 (12)	−0.0112 (9)	0.0121 (10)	−0.0003 (9)
C24	0.1051 (18)	0.0593 (12)	0.0550 (11)	0.0036 (12)	0.0132 (11)	0.0155 (9)
C22	0.0696 (13)	0.0760 (13)	0.0493 (10)	0.0074 (11)	−0.0063 (9)	−0.0037 (9)
C18	0.0812 (15)	0.0627 (12)	0.0703 (13)	−0.0092 (11)	−0.0067 (11)	−0.0155 (10)
C23	0.0920 (16)	0.0829 (15)	0.0454 (10)	0.0132 (13)	0.0023 (10)	0.0154 (10)
C2	0.0915 (16)	0.0515 (11)	0.0610 (11)	−0.0048 (10)	0.0037 (11)	0.0170 (9)
C3	0.0824 (16)	0.0615 (12)	0.0761 (14)	−0.0158 (11)	0.0176 (12)	0.0134 (11)
C15	0.0590 (13)	0.0600 (13)	0.134 (2)	−0.0097 (10)	−0.0191 (14)	0.0133 (14)
C17	0.106 (2)	0.0773 (16)	0.0917 (17)	−0.0128 (15)	−0.0253 (15)	−0.0245 (13)
C11	0.0917 (18)	0.0971 (18)	0.0797 (15)	0.0306 (15)	0.0149 (13)	−0.0223 (14)
C16	0.098 (2)	0.0653 (15)	0.127 (2)	−0.0177 (14)	−0.0437 (18)	−0.0097 (16)
C19	0.0838 (19)	0.0871 (19)	0.164 (3)	−0.0004 (15)	0.0523 (19)	0.0032 (19)

### Geometric parameters (Å, º)

**Table:**

O2—N1	1.4306 (17)	C13—C14	1.389 (3)
O2—C12	1.436 (2)	C9—H9A	0.9700
O3—C10	1.196 (2)	C9—H9B	0.9700
O1—C1	1.379 (2)	C14—C15	1.391 (3)
O1—C9	1.435 (2)	C21—C22	1.390 (2)
O4—C10	1.325 (2)	C21—H21	0.9300
O4—C11	1.446 (3)	C4—C3	1.378 (3)
N1—C20	1.426 (2)	C4—H4	0.9300
N1—C7	1.489 (2)	C24—C23	1.368 (3)
O5—C14	1.368 (3)	C24—H24	0.9300
O5—C19	1.419 (3)	C22—C23	1.374 (3)
C8—C10	1.524 (2)	C22—H22	0.9300
C8—C9	1.524 (2)	C18—C17	1.386 (3)
C8—C7	1.553 (2)	C18—H18	0.9300
C8—C12	1.558 (2)	C23—H23	0.9300
C20—C21	1.387 (2)	C2—C3	1.373 (3)
C20—C25	1.391 (2)	C2—H2	0.9300
C7—C6	1.510 (2)	C3—H3	0.9300
C7—H7	0.9800	C15—C16	1.369 (4)
C25—C24	1.382 (3)	C15—H15	0.9300
C25—H25	0.9300	C17—C16	1.361 (4)
C6—C1	1.385 (2)	C17—H17	0.9300
C6—C5	1.387 (3)	C11—H11A	0.9600
C12—C13	1.500 (2)	C11—H11B	0.9600
C12—H12	0.9800	C11—H11C	0.9600
C5—C4	1.382 (3)	C16—H16	0.9300
C5—H5	0.9300	C19—H19A	0.9600
C1—C2	1.394 (3)	C19—H19B	0.9600
C13—C18	1.384 (3)	C19—H19C	0.9600
			
N1—O2—C12	104.93 (11)	C8—C9—H9B	109.3
C1—O1—C9	111.21 (13)	H9A—C9—H9B	108.0
C10—O4—C11	116.32 (16)	O5—C14—C15	124.6 (2)
C20—N1—O2	111.71 (12)	O5—C14—C13	115.12 (18)
C20—N1—C7	117.71 (12)	C15—C14—C13	120.3 (2)
O2—N1—C7	105.14 (11)	C20—C21—C22	119.92 (18)
C14—O5—C19	118.8 (2)	C20—C21—H21	120.0
C10—C8—C9	110.07 (14)	C22—C21—H21	120.0
C10—C8—C7	113.09 (13)	C3—C4—C5	119.5 (2)
C9—C8—C7	110.18 (14)	C3—C4—H4	120.2
C10—C8—C12	110.94 (14)	C5—C4—H4	120.2
C9—C8—C12	111.05 (14)	C23—C24—C25	121.4 (2)
C7—C8—C12	101.27 (12)	C23—C24—H24	119.3
C21—C20—C25	119.05 (15)	C25—C24—H24	119.3
C21—C20—N1	119.60 (15)	C23—C22—C21	120.78 (19)
C25—C20—N1	120.90 (15)	C23—C22—H22	119.6
N1—C7—C6	111.29 (13)	C21—C22—H22	119.6
N1—C7—C8	105.62 (12)	C13—C18—C17	121.3 (2)
C6—C7—C8	113.73 (13)	C13—C18—H18	119.3
N1—C7—H7	108.7	C17—C18—H18	119.3
C6—C7—H7	108.7	C24—C23—C22	119.11 (18)
C8—C7—H7	108.7	C24—C23—H23	120.4
C24—C25—C20	119.77 (18)	C22—C23—H23	120.4
C24—C25—H25	120.1	C3—C2—C1	119.25 (19)
C20—C25—H25	120.1	C3—C2—H2	120.4
C1—C6—C5	118.08 (16)	C1—C2—H2	120.4
C1—C6—C7	121.14 (16)	C2—C3—C4	120.65 (19)
C5—C6—C7	120.63 (15)	C2—C3—H3	119.7
O2—C12—C13	108.84 (14)	C4—C3—H3	119.7
O2—C12—C8	101.94 (13)	C16—C15—C14	119.8 (3)
C13—C12—C8	117.07 (14)	C16—C15—H15	120.1
O2—C12—H12	109.5	C14—C15—H15	120.1
C13—C12—H12	109.5	C16—C17—C18	119.3 (3)
C8—C12—H12	109.5	C16—C17—H17	120.4
C4—C5—C6	121.29 (18)	C18—C17—H17	120.4
C4—C5—H5	119.4	O4—C11—H11A	109.5
C6—C5—H5	119.4	O4—C11—H11B	109.5
O1—C1—C6	120.62 (16)	H11A—C11—H11B	109.5
O1—C1—C2	118.22 (16)	O4—C11—H11C	109.5
C6—C1—C2	121.16 (18)	H11A—C11—H11C	109.5
C18—C13—C14	118.28 (19)	H11B—C11—H11C	109.5
C18—C13—C12	122.30 (19)	C17—C16—C15	121.1 (2)
C14—C13—C12	119.42 (18)	C17—C16—H16	119.5
O3—C10—O4	123.87 (18)	C15—C16—H16	119.5
O3—C10—C8	124.12 (17)	O5—C19—H19A	109.5
O4—C10—C8	112.00 (14)	O5—C19—H19B	109.5
O1—C9—C8	111.39 (14)	H19A—C19—H19B	109.5
O1—C9—H9A	109.3	O5—C19—H19C	109.5
C8—C9—H9A	109.3	H19A—C19—H19C	109.5
O1—C9—H9B	109.3	H19B—C19—H19C	109.5
			
C12—O2—N1—C20	−87.53 (14)	C8—C12—C13—C18	−88.9 (2)
C12—O2—N1—C7	41.24 (15)	O2—C12—C13—C14	−153.74 (16)
O2—N1—C20—C21	170.08 (14)	C8—C12—C13—C14	91.4 (2)
C7—N1—C20—C21	48.3 (2)	C11—O4—C10—O3	0.4 (3)
O2—N1—C20—C25	−17.7 (2)	C11—O4—C10—C8	179.75 (18)
C7—N1—C20—C25	−139.49 (17)	C9—C8—C10—O3	−14.9 (2)
C20—N1—C7—C6	−129.01 (15)	C7—C8—C10—O3	−138.62 (17)
O2—N1—C7—C6	105.90 (14)	C12—C8—C10—O3	108.40 (19)
C20—N1—C7—C8	107.13 (15)	C9—C8—C10—O4	165.77 (15)
O2—N1—C7—C8	−17.97 (15)	C7—C8—C10—O4	42.1 (2)
C10—C8—C7—N1	−128.15 (14)	C12—C8—C10—O4	−70.92 (18)
C9—C8—C7—N1	108.20 (15)	C1—O1—C9—C8	−64.7 (2)
C12—C8—C7—N1	−9.40 (15)	C10—C8—C9—O1	−71.94 (19)
C10—C8—C7—C6	109.53 (16)	C7—C8—C9—O1	53.44 (19)
C9—C8—C7—C6	−14.11 (19)	C12—C8—C9—O1	164.82 (15)
C12—C8—C7—C6	−131.72 (14)	C19—O5—C14—C15	−11.3 (3)
C21—C20—C25—C24	0.3 (3)	C19—O5—C14—C13	169.5 (2)
N1—C20—C25—C24	−171.90 (18)	C18—C13—C14—O5	−179.71 (18)
N1—C7—C6—C1	−134.52 (15)	C12—C13—C14—O5	0.0 (3)
C8—C7—C6—C1	−15.4 (2)	C18—C13—C14—C15	1.0 (3)
N1—C7—C6—C5	50.02 (19)	C12—C13—C14—C15	−179.30 (18)
C8—C7—C6—C5	169.15 (14)	C25—C20—C21—C22	0.7 (3)
N1—O2—C12—C13	−171.11 (12)	N1—C20—C21—C22	173.05 (17)
N1—O2—C12—C8	−46.80 (14)	C6—C5—C4—C3	−0.7 (3)
C10—C8—C12—O2	153.58 (13)	C20—C25—C24—C23	−1.0 (3)
C9—C8—C12—O2	−83.68 (16)	C20—C21—C22—C23	−1.1 (3)
C7—C8—C12—O2	33.30 (14)	C14—C13—C18—C17	−0.9 (3)
C10—C8—C12—C13	−87.82 (18)	C12—C13—C18—C17	179.4 (2)
C9—C8—C12—C13	34.9 (2)	C25—C24—C23—C22	0.6 (4)
C7—C8—C12—C13	151.90 (15)	C21—C22—C23—C24	0.5 (3)
C1—C6—C5—C4	−0.6 (3)	O1—C1—C2—C3	178.34 (19)
C7—C6—C5—C4	174.99 (16)	C6—C1—C2—C3	−1.0 (3)
C9—O1—C1—C6	33.7 (2)	C1—C2—C3—C4	−0.4 (3)
C9—O1—C1—C2	−145.58 (18)	C5—C4—C3—C2	1.2 (3)
C5—C6—C1—O1	−177.86 (15)	O5—C14—C15—C16	−179.8 (2)
C7—C6—C1—O1	6.6 (2)	C13—C14—C15—C16	−0.6 (3)
C5—C6—C1—C2	1.4 (3)	C13—C18—C17—C16	0.5 (4)
C7—C6—C1—C2	−174.15 (16)	C18—C17—C16—C15	−0.1 (4)
O2—C12—C13—C18	26.0 (2)	C14—C15—C16—C17	0.1 (4)

### Hydrogen-bond geometry (Å, º)

**Table:**

*D*—H···*A*	*D*—H	H···*A*	*D*···*A*	*D*—H···*A*
C7—H7···O4	0.98	2.33	2.803 (2)	109
C15—H15···O3^i^	0.93	2.42	3.285 (3)	155

Symmetry code: (i) −*x*+2, −*y*, −*z*+1.
